# Expression of POU-domain transcription factor, Oct-6, in schizophrenia, bipolar disorder and major depression

**DOI:** 10.1186/1471-244X-5-38

**Published:** 2005-10-24

**Authors:** Kirenjeet Ubhi, Jack Price

**Affiliations:** 1 Institute of Psychiatry, Kings College London, Denmark Hill, London, UK

## Abstract

**Background:**

The POU-domain transcription factor *Oct-6 *has been reported to be differentially expressed between schizophrenic and control post-mortem brains. In this study, we attempted to replicate this finding and to discover whether *Oct-6 *was also dysregulated in bipolar disorder and major depression.

**Methods:**

*Oct-6 *mRNA and protein expression were determined by in-situ hybridization and immunohistochemistry respectively in sections of post-mortem brain.

**Results:**

We did not observe any differences in *Oct-6 *expression between any of the groups under study. *Oct-6 *mRNA and protein was identically expressed in the hippocampal and cortical regions of most specimens in all groups, including controls.

**Conclusion:**

*Oct-6 *is, therefore, unlikely to be a specific marker for any psychological disorder; rather its expression in controls suggests that it is normally expressed in most adult brains.

## Background

The neurodevelopmental hypothesis of schizophrenia proposes that events occurring during foetal development, which adversely effect the development of the brain, may underlie the occurrence of schizophrenia in later life [1]. One approach to try to investigate this hypothesis has been to examine genes involved in the normal development of the brain and determine whether they are altered in schizophrenia.

The POU-domain homeobox transcription factors are one such family of genes involved in development. We have focused on *Oct-6 *(*SCIP/Tst-1/POU3f1*), a member of the POU-III subfamily. Much of the initial work on *Oct-6 *looked at its role in the peripheral nervous system, in particular its role in Schwann cell development, where it is required for the timely initiation of myelination [2]. *Oct-6 *has also been shown to be expressed in postmitotic neurons of the developing rodent telencephalon as they migrate from the ventricular to the intermediate zone, and is particularly associated with those destined for cortical layers II/III and V. *Oct-6 *is expressed in these cortical laminae and in the CA1 region of the adult hippocampus [3,4], although some rodent data has suggested that all *Oct-6 *expression is eventually lost with age [5].

A previous open study has reported the expression of OCT-6 protein in the frontal and temporal lobes of patients diagnosed with schizophrenia but its absence from matched controls, suggesting it may be a putative biological marker for schizophrenia [6]. Affective disorders such as bipolar disorder and major depression share many of the symptoms seen in schizophrenia (DSM-IV) [7], and it has been suggested that a common pathology may underlie schizophrenia and bipolar disorder [8-10]. The potential importance of POU factors in affective disorders was highlighted by a study by Stopkova et al (2004) who reported that a polymorphism within the PIK3C3 promoter, linked to a subset of schizophrenic and bipolar patients. Stopkova et al (2004) suggest that the -432C→T polymorphism, occurring as it does within an octamer binding site (the DNA motif recognised by POU domain transcription factors), may affect POU protein binding to this region [11]. Expression assays in neural stem cell lines have shown that *Oct-6*-induced expression of the reporter is indeed decreased by the presence of this polymorphism (personal communication, Dafe Uwanogho).

We aimed to replicate the initial finding of *Oct-6 *expression in the brains of patients diagnosed with schizophrenia and to extend this finding to bipolar disorder and major depression in order to determine whether *Oct-6 *expression was specific to schizophrenia or a more general marker of psychosis.

## Methods

### Tissue acquisition

A total of 60 subjects from the Stanley Foundation Neuropathology Consortium were used in these studies. This set consists of 15 samples from patients with schizophrenia, 15 with major depressive disorder, 15 with bipolar disorder, and 15 matched controls patients. A detailed description of this collection has been published [12], and a summary of subject characteristics is shown in Table 1.

**Table 1:** Summaries of demographic, clinical and histological information of schizophrenic, bipolar, major depression and control cases.

Demographics	Group			
	Controls n = 15	Schizophrenic n = 15	Bipolar disorder n = 15	Major Depression n = 15

AGE (years, mean ± SD)	48.1 ± 10.7	44.5 ± 13.1	42.3 ± 11.7	46.5 ± 9.3
GENDER	9M, 6F	9M, 6F	9M, 6F	9M, 6F
POSTMORTEM INTERVAL (hours, mean ± SD)	23.7 ± 9.9	33.7 ± 14.6	32.5 ± 16.1	27.5 ± 10.7
CAUSE OF DEATH				
CPD	13	7	11	8
Accident	2	0	0	2
Pneumonia	0	0	1	1
Suicide	0	7	1	1
Other	0	1	2	3
pH (mean ± SD)	6.3 ± 0.2	6.2 ± 0.3	6.2 ± 0.2	6.1 ± 0.3
FIXATION (months, mean ± SD)	4.40 ± 3.9	11.20 ± 8.5	9.67 ± 3.6	8.33 ± 6.6
*STORAGE OF FROZEN SECTIONS *(days, mean ± S D)	621 ± 172.3	434 ± 290.0	338 ± 234.2	621 ± 233.1
*BRAIN HEMISPHERE*	7R:8L	6R:9L	8R:7L	6R:9L
DURATION OF ILLNESS (years, mean ± SD)	0 ± 0	21.3 ± 11.4	20.1 ± 9.7	12.7 ± 11.1
LIFETIME ANTIPSYCHOTIC^a ^DOSE (mg) (min-median-max)	0-0-0	0- 35 000- 200 000	0- 7 500- 60 000	0-0-0

a = lifetime quantity of fluphenazine or equivalent

CPD = cardiac pulmonary disease

Cryostat and paraffin sectioned slides were provided by the Stanley Foundation. They were stored at -80°C and room temperature, respectively, until use. The sections were coded, the investigator had no knowledge of the disease state of the tissue and the experiments were conducted 'blind'.

### Antibodies

Anti-Oct-6 antibody 1 : rabbit polyclonal against the N-terminal region of mouse Oct-6 protein (98% homology in human), hereafter referred to as anti-Oct-6 (N). Antibody characterisation and specificity has been previously described [4].

Anti-Oct-6 antibody 2 : rabbit polyclonal against full-length Oct-6 purified from baculovirus infected Sf9 cells hereafter referred to as Anti-Oct-6(FL). Antibody characterisation and specificity has previously been described [13].

### Paraffin sections

Slides were dewaxed using xylene, rehydrated and endogenous peroxidase activity was blocked by washing in 0.3% H_2_O_2_. Following PBS (phosphate buffered saline) washes, slides were incubated in normal swine serum (1:10 in PBS; Dakocytomation) for 30 mins then incubated at 4°C overnight in Anti-Oct-6(N) (1:500). Slides were then incubated in anti-rabbit secondary (1:500 in PBS; Dakocytomation) for 2 hours. Slides were washed then incubated in ABComplex (Dakocytomation) for a further 2 hours in the dark. Colour was developed using DAB (3, 3'-diaminobenzidine, Vector Laboratories). The reaction took 5–10 minutes and was considered complete when a brown colour was observed on the slides. When suitable colour had developed, the DAB reaction was stopped by washing slides in dH_2_O, slides were air dried and mounted using Faramount aqueous mounting medium (Dakocytomation).

### Frozen sections

Slides were post fixed for 10 minutes in acetone (-20°C) for 10 minutes, air dried and washed in PBS (4°C) for 5 minutes and 0.3% H_2_O_2 _for 10 minutes. Slides were blocked for 30 minutes in a 1:10 (in PBS) solution of Swine serum (Dakocytomation) and incubated overnight at room temperature in anti-Oct-6(FL) (1:3000). Slides were washed then incubated in anti- rabbit secondary antibody (1:500, Dakocytomation) for 2 hours. Signal amplification and visualization using ABC and DAB was as described above.

### In Situ Hybridization

In situ hybridization was conducted using a 1.6 kb DIG labelled probe complementary to the 3' untranslated region of the Oct-6 gene.

Forward primer: GTGGTGGTGGTGGTGGTGTGTGACGGG

Reverse primer: ACAGCCCTGGGGTACATGTTTATGTGAGTAATAAAAT

Amplicon: 1646 bp

Slides were placed in 4% fomaldehyde at room temperature for 10 mins then washed in DEPC-PBS then in 0.1 M triethanolamine, pH 8.0/acetic anhydride, 400: 1 (vol: vol), on a stir plate for 10 min. Slides were then incubated in pre-hybridization solution (50% formamide, 5 × SSC, 5 × Denharts, 250 μg/ml bakers yeast RNA and 500 μg/ml Herring sperm DNA) for 2–4 hrs at room temperature in a humidified chamber. Slides were incubated in hybridization solution (pre-hybridization solution with probe added at a typical concentration of 1:500), covered with a coverslip and incubated at 60°C for approximately 18 hours. Sense-strand probes were used as specificity controls for hybridization and revealed no binding.

Coverslips were removed by washing in 5 × SSC at 65°C, then the slides were washed in 2 × SSC at 65°C for 30 mins, 0.2 × SSC at 65°C for 30 mins, then in Buffer 1 (0.1 M Tris pH7.5, 0.15 M NaCl) for 5 mins then incubated in block (10% heat inactivated sheep serum in Buffer 1) at room temperature for 1 hour then finally incubated in alkaline phosphatase-conjugated sheep anti-DIG antibody (Roche) at room temperature for 4 hours. Following washed in Buffer 1 and Buffer 3 (0.1 M Tris pH9.5, 0.1 M NaCl, 50 mM MgCl_2_, 2 mM levamisole), hybridization was visualized by enzyme catalysed colour reaction using nitro blue tetrazolium salt (NBT) and 5-bromo-4-chloro-3-indolyl phosphate (BCIP) (Roche). Reaction was stopped in PBS and then water, slides were air-dried and mounted using Faramount aqueous mounting medium (Dakocytomation).

### Image analysis

All sections were analysed with a Nikon light microscope (Eclipse 600) with Lucia 4.0 image analysis software.

## Results

### OCT-6 immunoreactivity in paraffin sections

**Figure 1:**
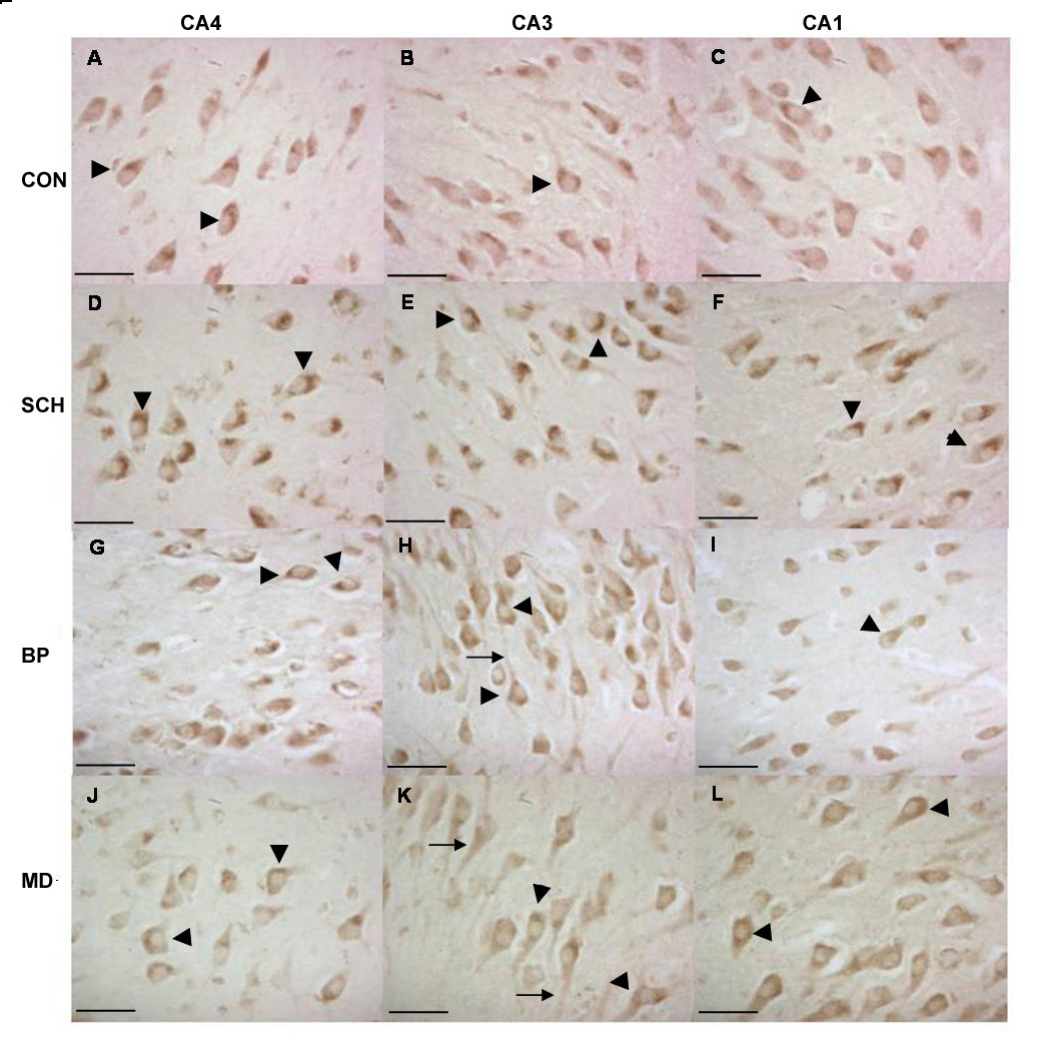
**Oct-6 Immunoreactivity in Paraffin-embedded Tissue from Control, Schizophrenic, Bipolar Disorder and Major Depression. **Oct-6 immunoreactivity was observed in hippocampal subfields CA4, CA3 and CA1 in controls (CON A – C), schizophrenics (SCH D – F), bipolar disorder (BP G – I) and major depression (MD J – L). Oct-6 immunoreactivity was observed in groups in a cytoplasmic/perinuclear fashion (arrowheads). Oct-6-positive cells had the morphological appearance of pyramidal neurons. Immunoreactivity is visible in the apical dendrites of some neurons (arrows in (H) and (K)) Scale bars = 50 μm

We attempted to detect OCT-6 immunoreactivity in hippocampus and cortex in post-mortem human brains. We performed immunohistochemistry on a series of 22 paraffin sections from the hippocampal formation taken from a total of 20 brains from all four groups under study (schizophrenia (n= 9), major depression (n = 3), bipolar disorder (n = 3), and control (n = 5)), using the antibody (Anti-Oct-6 (N)) and staining procedure as in the previous study [6]. In each case, we found OCT-6 expression in all fields (CA4, CA3 and CA1) of the hippocampal formation (Figure 1). OCT-6 immunoreactivity was restricted to large cells with the morphology of pyramidal neurons and appeared to be localised to the perinuclear region of the cytoplasm. There was no distinguishable difference in intensity of staining between different hippocampal fields.

**Figure 2:**
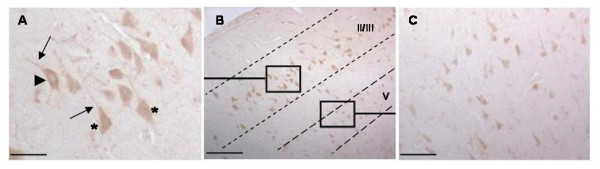
**Oct-6 Immunoreactivity in the Cerebral Cortex**. Oct-6 immunoreactivity in the cerebral cortex of a schizophrenic patient. Staining was observed in a layer-specific pattern restricted to layers II/III and V (B) seen at higher magnification in (A) & (C). Oct-6-positive cells had the morphological characteristics of pyramidal neurones. In general, POU3F1 immunoreactivity was dispersed throughout the cell (asterisks in A), though some did have the cytoplasmic/perinuclear localization noted in the hippocampus (arrowheads in A). Immunoreactivity was also observed in the apical dendrites (arrows). Cells in layer V (C) appeared considerably smaller than those in layer II/III (A). A similar cortical expression was observed in all groups. Scale bars: (B) = 200 μm (A & C) = 50 μm

Immunoreactivity was also noted in the cerebral cortex where it assumed a layer-specific pattern, appearing restricted to pyramidal neurons of layers II/III and V (Figure 2). Cells in layer II/III were more intensely stained than those in layer V. OCT-6 expression in all cortical neurons generally appeared to be dispersed throughout the cell, although a few neurons in layer II/III were noted to have the cytoplasmic/perinuclear localization seen in hippocampal cells (Figure 2A (arrowhead)). This laminar distribution in the neocortex is consistent with the pattern described in adult rodents [3].

Thus, in contrast to the previous study [6], we observed OCT-6 immunoreactivity in hippocampal field CA1 and were unable to detect any difference in OCT-6 expression between control and schizophrenic specimens, as all the control specimens we examined (n = 5) displayed Oct-6 immunoreactivity.

### OCT-6 immunoreactivity in frozen sections

The immunoreactivity in the paraffin sections was predominantly cytoplasmic. Though this has been reported in earlier studies [6, 14], it is nonetheless noteworthy since *Oct-6 *being a transcription factor would be expected to be nuclear. It was deemed possible that the fixation may be affecting antibody penetration. In order to investigate this further, immunohistochemistry was conducted on fresh frozen sections. We were unable to observe any immunoreactivity in unfixed specimens using the anti-Oct-6 (N) antibody. We were, however, able to observe robust, reproducible staining using the anti-Oct-6(FL) antibody. Oct-6 immunoreactivity was observed in a subset of cells in hippocampal fields CA4, CA3, CA1 in all groups (Figure 4). As with the paraffin sections, staining appeared restricted to pyramidal neurons. Not all specimens had Oct-6 immunoreactive cells, but in each group the majority of specimens were positive (6 of 7 control specimens, 8 of 8 schizophrenic specimens, 6 of 7 bipolar specimens and 7 of 8 major depression specimens). There was no significant difference in the proportion of positive specimens between groups.

In all positive cases staining was evident throughout the hippocampal formation from CA4 to CA1, but different fields showed different degrees of immunoreactivity with the most intense staining generally appearing in CA1.

**Figure 3:**
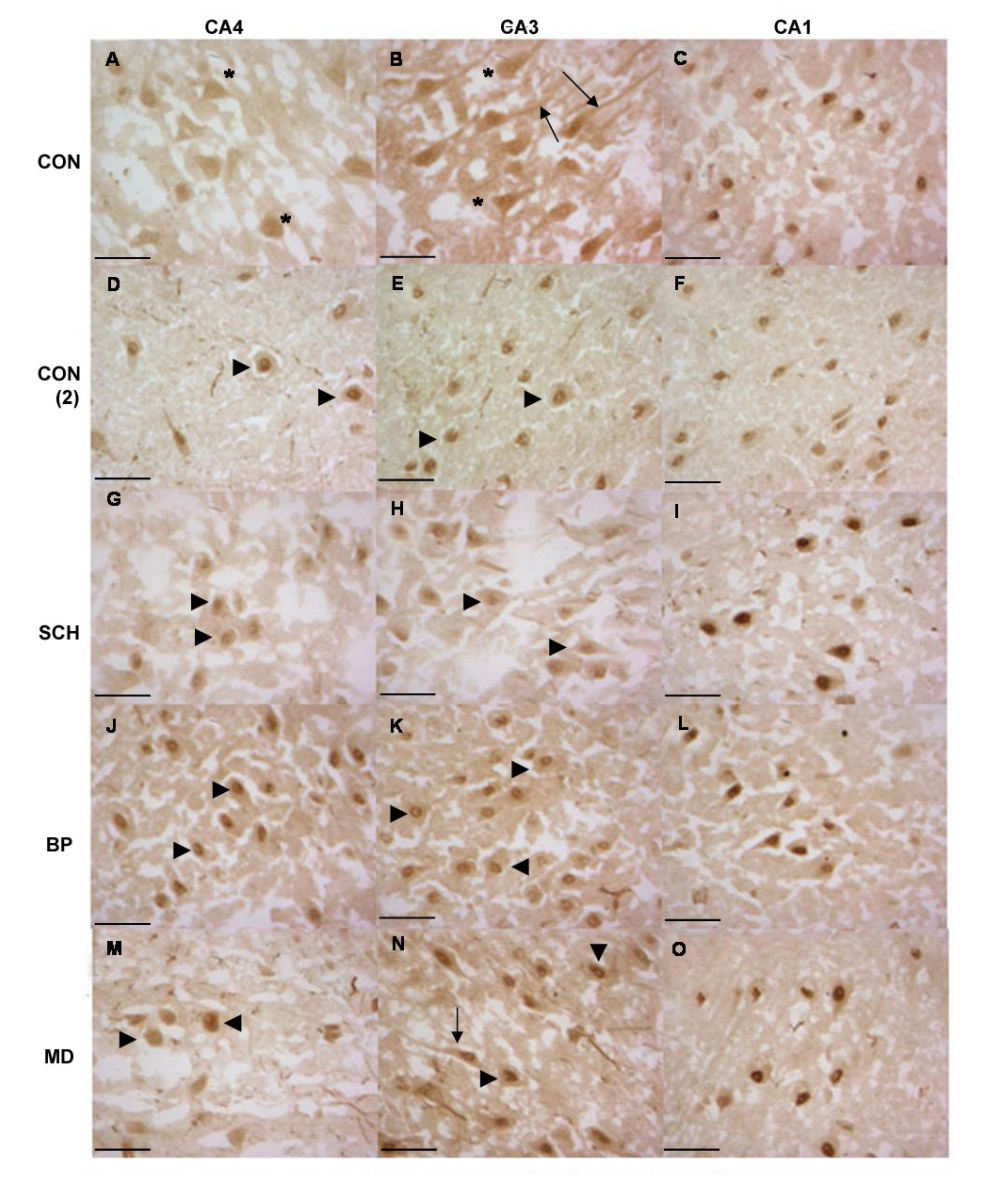
**Oct-6 Immunoreactivity in Fresh-frozen Tissue from Control, Schizophrenic, Bipolar Disorder and Major Depression. **Oct-6 immunoreactivity was observed in all groups (CON A – C and CON (2) D- F), schizophrenics (SCH G – I), bipolar disorder (BP J – L) and major depression (MD M – O) and did not differ between groups or in comparison to controls. Oct-6-positive cells had the morphological appearance of pyramidal neurons. In the CA4 and CA3 regions immunoreactivity generally took on a nuclear localization (arrowheads) thought in some cases a more dispersed pattern of expression was noted (asterisks). Oct-6 immunoreactivity was also noted the apical dendrites of some cells (arrows). Though staining was robust throughout, it appeared stronger in the CA1 region (C, F, I, L & O), in all cases Oct-6 immunoreactivity in the CA1 was nuclear. Scale bars = 50 μm

Differences in staining localization between the different hippocampal fields were apparent. In some specimens, Oct-6 immunoreactivity in CA4 and CA3 was present throughout the neuron cell body (Figure 3A (asterisks), whilst in others it was predominantly nuclear (Figure 3D, E, J and 3K (arrows). Immunoreactivity was also noted in the apical dendrites of some cells (Figure 3B (arrowheads). In all cases the intense staining seen in the CA1 region was predominantly nuclear (Figure 3C, F, I, L and 3O). These differences in localization did not appear to be particularly associated with any of the four groups under study.

### In situ hybridization (ISH)

**Figure 4:**
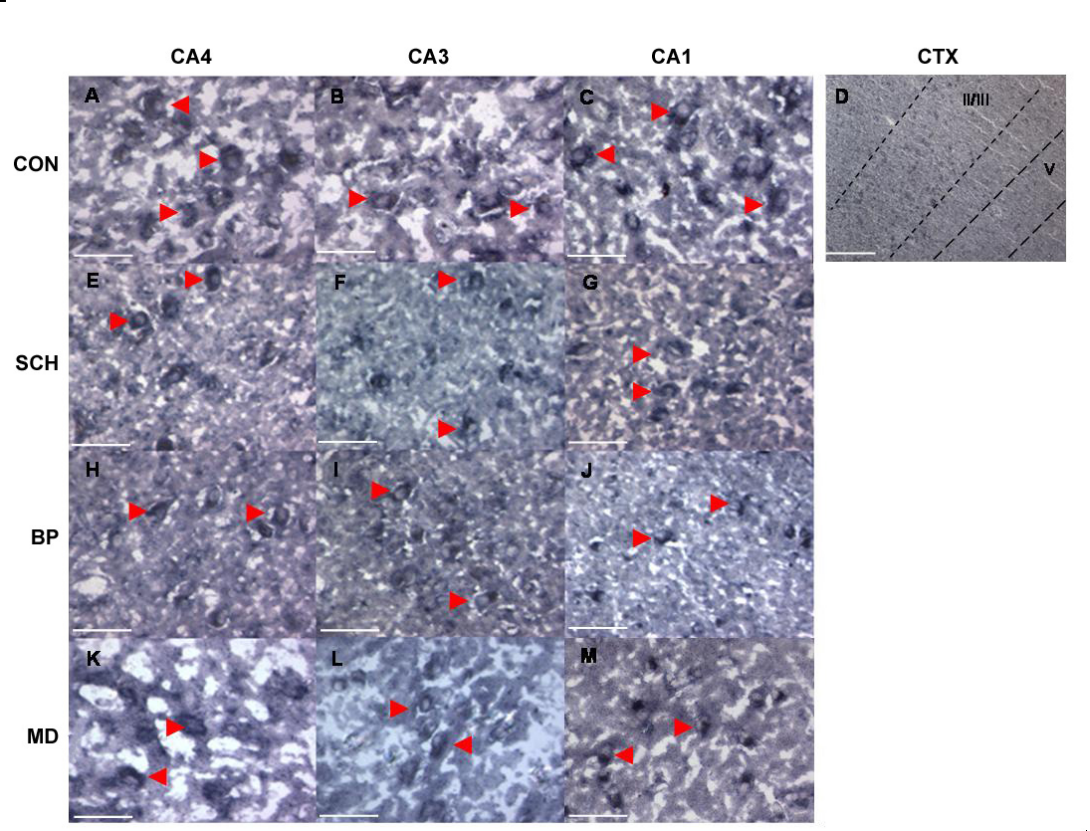
***Oct-6 *mRNA expression in Control, Schizophrenic, Bipolar Disorder and Major Depression Patients. ***Oct-6 *mRNA expression (arrowheads) was observed in hippocampal fields CA4 (A, E, H & K), CA3 (B, F, I & L) and CA1 (C, G, J and M) in all groups including controls (controls = CON, schizophrenics = SCH bipolar disorder = BP and major depression = MD). No qualitative differences were noted in hybridization signal between different regions or groups. *Oct-6 *mRNA was observed in a layer-specific pattern in the cortical layers II/III and V (D). Scale bars: (D) = 500 μm, all others = 50 μm

In order to determine areas of *Oct-6 *gene expression, ISH was performed using a probe complementary to the 3'UTR of the *Oct-6 *gene. *Oct-6 *mRNA expression was observed in the cortex and all hippocampal fields in specimens from all four groups (Figure 4). As with immunoreactivity, the majority of specimens in each group were positive (9 of 14 control specimens, 9 of 15 schizophrenic specimens, 9 of 14 bipolar specimens and 9 of 14 major depression specimens). Thus, the proportion of positive specimens was almost identical in each group.

Although levels of immunoreactivity varied between hippocampal fields, the ISH signal appeared to be at similar levels throughout, being equally intense in the CA4 as the CA1 cells. There were some positive cells in cortical regions, but intensity here was much reduced. In the cortex the expression of *Oct-6 *mRNA was confined to layers II/III and V, similar to the Oct-6 immunoreactivity described above.

### Statistical analysis of confounding variables

Kruskal-Wallis non-parametric tests revealed significant differences between groups with regards to the length of time tissue had spent either in formalin or in frozen storage (df = 3 p = 0.017 and df = 3, p = 0.005 respectively) in each case the control samples had spent shorter amounts of time in each.

## Discussion

In this study, we sought to reproduce the previously reported finding that OCT-6 was expressed by pyramidal neurons in the hippocampus of schizophrenic brains, but not in controls [6]. We attempted to extend this finding to include bipolar disorder and major depression. Using immunoreactivity and ISH we observed *Oct-6 *expression in all three disease states, but unlike the previous study we also observed expression in the control group. So while we can confirm that *Oct-6 *is indeed expressed by pyramidal neurons in the schizophrenic brain (and in bipolar disorder and major depression), there is no observable difference from controls.

Each of the three approaches use in this study gave subtly different results in terms of the localisation of expression and the proportions of specimens that express *Oct-6*. These differences most likely reflect differences in the techniques themselves, such as fixation. Note also that the paraffin and frozen sections were from different sides of the brain randomly chosen to limit laterality. Nonetheless, no differences between the disease groups and controls emerge with any technique.

The paraffin immunohistochemistry indicated that *Oct-6 *was expressed in all specimens, regardless of group. The fresh frozen immunohistochemistry and ISH gave positive results in approximately 90% of cases. Therefore, either there are a small number of *Oct-6 *negative brains, or the fresh frozen sections gave occasional false negatives. A second difference that emerges is that while ISH detected a signal of similar intensity in all hippocampal fields, the immunohistochemistry appeared to give a stronger signal in CA1, though clear staining was observed in all hippocampal fields. It is possible that this difference in expression could be due OCT-6 stability or translational efficiency in the CA1. This is currently under investigation and we have observed evidence of translational control of *Oct-6 *in studies in vitro (unpublished observations).

The final difference to emerge from our studies was the difference in localisation of immunoreactivity between paraffin and frozen sections. In paraffin sections, the immunoreactivity was exclusively cytoplasmic, whilst in frozen sections it was predominantly nuclear. The cytoplasmic expression was somewhat unexpected for a transcription factor, but not unprecedented. Some transcription factors are regulated by sequestration in the cytoplasm, NFêB for example [15], and a cytoplasmic localisation for *Oct-6 *has previously been reported in Schwann cells. It was also possible; however, that the absence of nuclear staining was an artefact of fixation, and the fact that we observe nuclear staining in the fresh frozen sections supported this notion.

It is important to note that, despite the fact that no definite statements regarding the cellular localization of Oct-6 immunoreactivity can be made, Oct-6 immunoreactivity was observed in similar regions all groups including controls, regardless of the fixation method used. The expression of *Oct-6 *mRNA coincided with protein expression and again was observed in all groups including controls. Taken as a whole, the data from this study strongly argue against *Oct-6 *being a marker of schizophrenia or any other psychiatric condition.

Our overall observations are consistent with *Oct-6 *expression seen in the rodent [3,4], with the one difference that in the adult rodent *Oct-6 *expression becomes restricted to the CA1 field of the hippocampus and layer V of the cerebral cortex with age, whereas we see expression in all fields of the human hippocampal formation. It is possible that the restriction may have yet to occur in the brains we investigated, but in rodents the adult pattern is observed by postnatal day 30 [3] while our specimens had an average age of 45, so this explanation is unlikely.

It is unclear why we have been unable to reproduce the earlier result. The few negative specimens seen with fresh frozen sections suggest that *Oct-6*^-ve ^specimens may occur and thus it is conceivable that all the controls in the previous study were genuinely *Oct-6*^-ve^. Unfortunately, we do not have access to these earlier specimens in order to verify this point. Another possibility is that the specimens used by Ilia et al (2002) [6] were different in terms of some other significant uncontrolled variable. We have compared the two groups of specimens and have been unable to identify such a variable. A comparison of the ages of control specimens, for example, indicated no difference between the two (df = 23, p= 0.0913).

## Conclusion

The expression of *Oct-6*, both mRNA and protein, in the majority of samples including controls would seem to suggest that the expression we observe is a reflection of normal Oct-6 expression in the adult. We conclude therefore that *Oct-6*, rather than being a specific biological marker of schizophrenia, or any other psychotic state, is likely to be normally expressed in the adult brain.

## Competing interests

The author(s) declare that they have no competing interests.

## Authors' contributions

All experimental work and analysis was carried out by KU. JP contributed to the preparation of the final manuscript.

## Pre-publication history

The pre-publication history for this paper can be accessed here:


